# Effect of pedicle screw augmentation with a self-curing elastomeric material under cranio-caudal cyclic loading—a cadaveric biomechanical study

**DOI:** 10.1186/s13018-018-0958-z

**Published:** 2018-10-11

**Authors:** Werner Schmoelz, Alexander Keiler, Marko Konschake, Richard A Lindtner, Alessandro Gasbarrini

**Affiliations:** 1 Department of Trauma Surgery, Medical University of Innsbruck, Anichstraße 35, 6020 Innsbruck, Austria; 2 Department of Anatomy, Histology and Embryology, Medical University of Innsbruck, Innsbruck, Austria; 3 Instituto Orthopedic Rizzoli, Bologna, Italy

**Keywords:** Pedicle screw anchorage, Screw augmentation, PMMA, Silicone, Elastoplasty, In situ screw augmentation

## Abstract

**Background:**

Pedicle screws can be augmented with polymethylmethacrylate (PMMA) cement through cannulated and fenestrated pedicle screws to improve screw anchorage. To overcome the drawbacks of PMMA, a modified augmentation technique applying a self-curing elastomeric material into a balloon-created cavity prior to screw insertion was developed and evaluated. The aim of the study was to compare the effect of the established and novel augmentation technique on pedicle screw anchorage in a biomechanical in vitro experiment.

**Methods:**

In ten lumbar vertebral bodies, the right pedicles were instrumented with monoaxial cannulated and fenestrated pedicle screws and augmented in situ with 2 ml PMMA. The left pedicles were instrumented with monoaxial cannulated pedicle screws. Prior to left screw insertion, a balloon cavity was created and filled with 3 ml of self-curing elastomer (silicone). Each screw was subjected to a cranio-caudal cyclic load starting from − 50 to 50 N while the upper load was increased by 5 N every 100 load cycles until loosening or 11,000 cycles (600 N). After cyclic loading, a pullout test of the screws was conducted.

**Results:**

The mean cycles to screw loosening were 9824 ± 1982 and 7401 ± 1644 for the elastomer and PMMA group, respectively (*P* = 0.012). The post-cycling pullout test of the loosened screws showed differences in the failure mode and failure load, with predominantly pedicle/vertebrae fractures in the PMMA group (1188.6 N ± 288.1) and screw pullout through the pedicle (671.3 N ± 332.1) in the elastomer group.

**Conclusion:**

The modified pedicle screw augmentation technique involving a balloon cavity creation and a self-curing elastomeric silicone resulted in a significantly improved pedicle screw anchorage under cyclic cranio-caudal loading when compared to conventional in situ PMMA augmentation.

## Background

Posterior implant systems are used for stabilization and fixation of the human thoracic and lumbar spine if the load-bearing capacity is lost by injury, disease, or degeneration [1]. In selected cases, such as patients with reduced bone quality or in revision spine surgery, anchorage and load bearing of pedicle screws are diminished [2], and augmentation of transpedicular screws is recommended [2–7]. In general, pedicle screws can be augmented with two different techniques. The so-called in situ augmentation applies a specifically designed cannulated and fenestrated pedicle screw and carries out augmentation through the cannulated pedicle screw after screw placement. Alternatively, the augmentation can be conducted prior to screw placement with a standard pedicle screw being placed in the non-cured augmentation material. In order to better control the flow of the augmentation material, a cavity may be created by cutting a screw thread or by kyphoplasty balloon inflation prior to augmentation [5, 8–10].

Although polymethylmethacrylate (PMMA) has been used for many years—at the beginning for reconstructive surgery, more recently as augmentation material in several conditions—the application can have negative side effects. Due to its exothermic curing behavior, the temperature at the interface increases substantially, and concerns for thermal bone necrosis were raised [11–13]. Other drawbacks include the lack of osteoconductivity [14–16], the limited time frame for processing [17], and the still not entirely investigated interaction between PMMA and the surrounding tissue, as in some cases a toxic property of the monomer (methyl methacrylate) has been reported in the literature [18–22]. Bone cements are extensively employed in orthopedics for joint arthroplasty. However, implant failure in the form of aseptic loosening is known to occur after long-term use [23]. Laboratory studies showed a decrease in molecular weight and hydrolysis of PMMA associated with long-term implantation [24] while the stress-strain behavior of the PMMA/bone composite is affected by the polymerization shrinkage during curing [25]. Because PMMA was already in clinical use when the FDA gained regulatory authority over medical devices, its approval was grandfathered by the FDA. Current testing of PMMA is conducted in its fully cured state and not in the state of the application to the human body (ASTM standard) [26].

Consequently, the emphasis was put on the development of alternative augmentation materials—for example, calcium-phosphate cement, calcium-sulfate cement, or silicone. Calcium-based cements are osteoconductive and osteoinductive [27] but have drawbacks such as long curing time or early resorption [28]. The use of silicones in the medical field has constantly increased since the 1960s, and nowadays, they are a thoroughly tested and important biomaterial with well-documented biocompatible and biodurable properties [29, 30].

With the development of a medical grade, injectable, self-curing elastomeric polymer with silicone basis, a new alternative material is available for vertebral augmentation. The silicone is intended to be osteoconductive and non-hazardous to the surrounding tissue, showing a non-exothermic curing [31]. Although silicones have been used and tested in the medical field for some decades, the augmentation of implants with silicones has not been investigated yet and represents the application of a well-known material in a new field [29].

The purpose of the present study was to compare the fixation strength of in situ PMMA-augmented fenestrated pedicle screws with that of standard pedicle screws inserted in a balloon-created cavity filled with an injectable silicone-based polymer. We hypothesized that the number of cranio-caudal load cycles until screw loosening does not differ between in situ PMMA-augmented screws and balloon cavity-augmented silicone screws.

## Methods

**Fig. 1:**
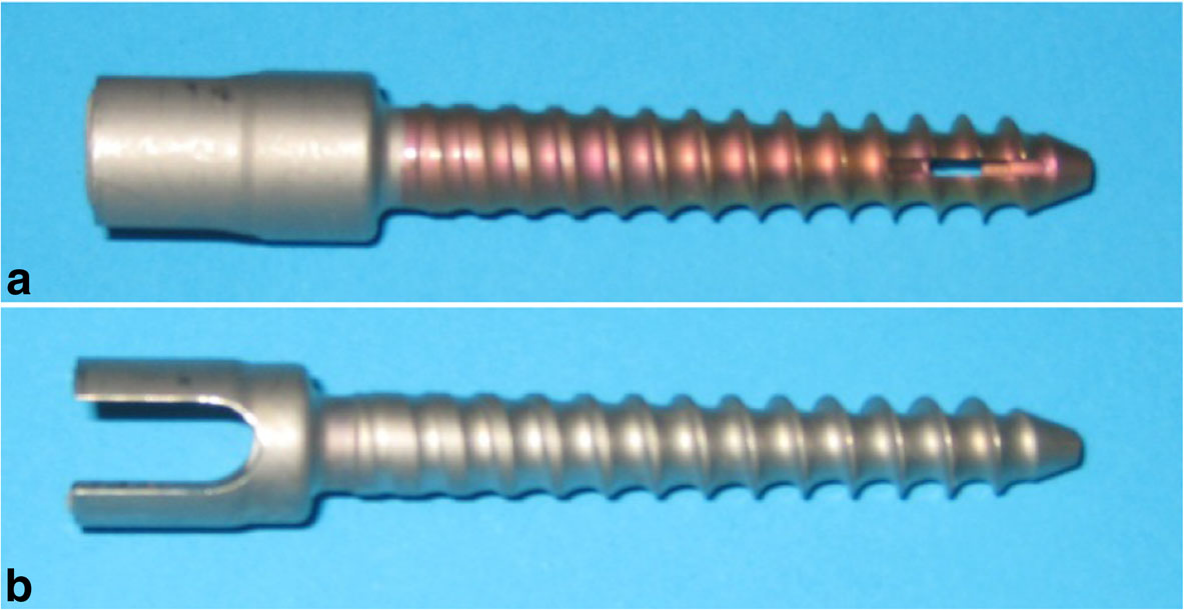
Screw types used. **a** 5.5 × 35 mm cannulated and fenestrated monoaxial screw. **b** 5.5 × 35 mm monoaxial cannulated screw

Ten lumbar vertebrae (L1–L5) from human donors were used for biomechanical testing. The bodies were donated by people who had given their informed consent for their use for scientific and educational purposes prior to death [32–34]. The specimens were double shrink-wrapped and frozen at − 20 °C. Prior to testing, a quantitative computed tomography (LightSpeed VCT 64; GE Healthcare, Waukesha, WI) was performed to rule out bony pathologies and to determine the bone mineral density via a European Forearm Phantom calibration. Specimens had an average age of 77.7 ± 8.7 years and an average BMD of 92.1 ± 33.6 mg/cm^3^. Specimens were thawed overnight at 6 °C, prepared, and implanted at room temperature. Monoaxial pedicle screws of identical size and thread geometry were used for the left to right comparison (S^4^, BBraun, Milan, Italy). For in situ PMMA augmentation, the screws were cannulated and fenestrated, while for balloon cavity augmentation, the screws were only cannulated (Fig. 1a. b).

Left pedicle screws were augmented with medical silicone (VK100, BONWRx, Lansing, MI, USA) using the kyphoplasty technique (inflatable balloon 15 mm, Tsunami SRL, Medolla, Italy) to create a cavity which was filled with 3 ml of silicone prior to screw placement. After this, a cannulated pedicle screw was inserted into the cured silicone.

Into the right pedicles, cannulated and fenestrated pedicle screws were implanted and in situ augmented with 2 ml of PMMA cement (Osteofix, Tsunami SRL, Medolla, Italy).

Isolated single vertebral bodies were embedded in PMMA (Technovit 3040, Heraeus Kulzer GmbH, Wehrheim, Germany) for fixation purpose in the testing machine, and an axial X-ray was taken to document the cement distribution.

**Fig. 2:**
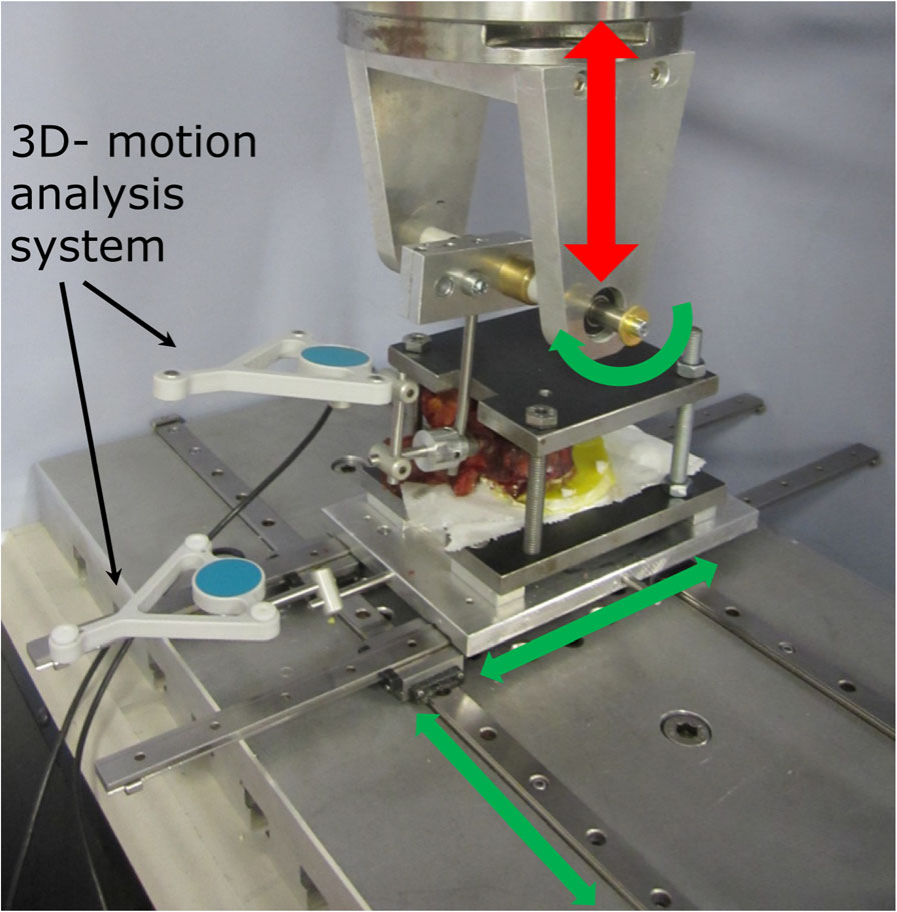
Test setup of the material testing machine. Green arrows indicate the degrees of freedom while the cranio-caudal load (red arrow) is applied. A 3D motion analysis system is fixed to the base plate as well as the pedicle screw head

Cyclic loading in cranio-caudal direction was conducted in a servohydraulic biaxial material testing machine (858 Mini Bionix II, MTS, Eden Prairie, MN, USA). Specimens were fixed on an *x*-*y* plane-bearing table, and a straight rod (Ø 5.5 mm) was connected to the pedicle screw. Axial loading was conducted with a lever arm of 15 mm to the pedicle screw head [35]. A 3D motion analysis system (Zebris, Winbiomechanics, Isny, Germany) was mounted to the pedicle screw and to the base plate to measure the relative motion of the pedicle screw head to the fixed vertebra (Fig. 2).

Each pedicle screw was initially cycled with 50 N in tension and 50 N in compression (speed 5 mm/s), with an increase of compressive load by 5 N every 100th cycle for a total of 11,000 cycles (600 N compressive loading). The cyclic protocol was terminated after 10 mm axial displacement of the machine crosshead.

For post-test data analysis, the motion of the screw head relative to the vertebra was evaluated. Screw loosening was defined as an absolute angular tilt of more than 8° or an increase in angular motion of more than 1° within one load step (100 cycles).

After cyclic loading, each pedicle screw was subjected to a pullout test in the direction of the screw axis. Pullout testing was conducted in a material testing machine (858 Mini Bionix II, MTS, Eden Prairie, MN, USA) with a speed of 10 mm/min. During all testing, the displacement and force at the actuator were recorded with 100 Hz.

### Statistical analysis

Statistical analysis was carried out using SPSS software package (version 21.0, SPSS, Chicago, IL, USA). Data are given as mean ± SD. Paired *t* tests were applied for comparisons between the two augmentation techniques (screws inserted into the left pedicles (silicone) vs. screws inserted into the right pedicles (PMMA)). Level of significance was set to a value of *P* < 0.05.

## Results

One in situ augmented pedicle screw placed in an L1 vertebra breached the pedicle after implantation. Therefore, this vertebra was excluded from the data analysis resulting in a total of nine vertebrae with left-right comparisons of the two augmentation techniques.

### Cyclic loading

All in situ PMMA-augmented screws loosened by caudal screw cutout through the pedicle. In the balloon cavity silicone-augmented group, only two screws loosened by caudal screw cutout through the pedicle. Two screws showed a loosening of the screw inside the intact pedicle, and five screws did not reach the predefined failure criteria.

**Fig. 3:**
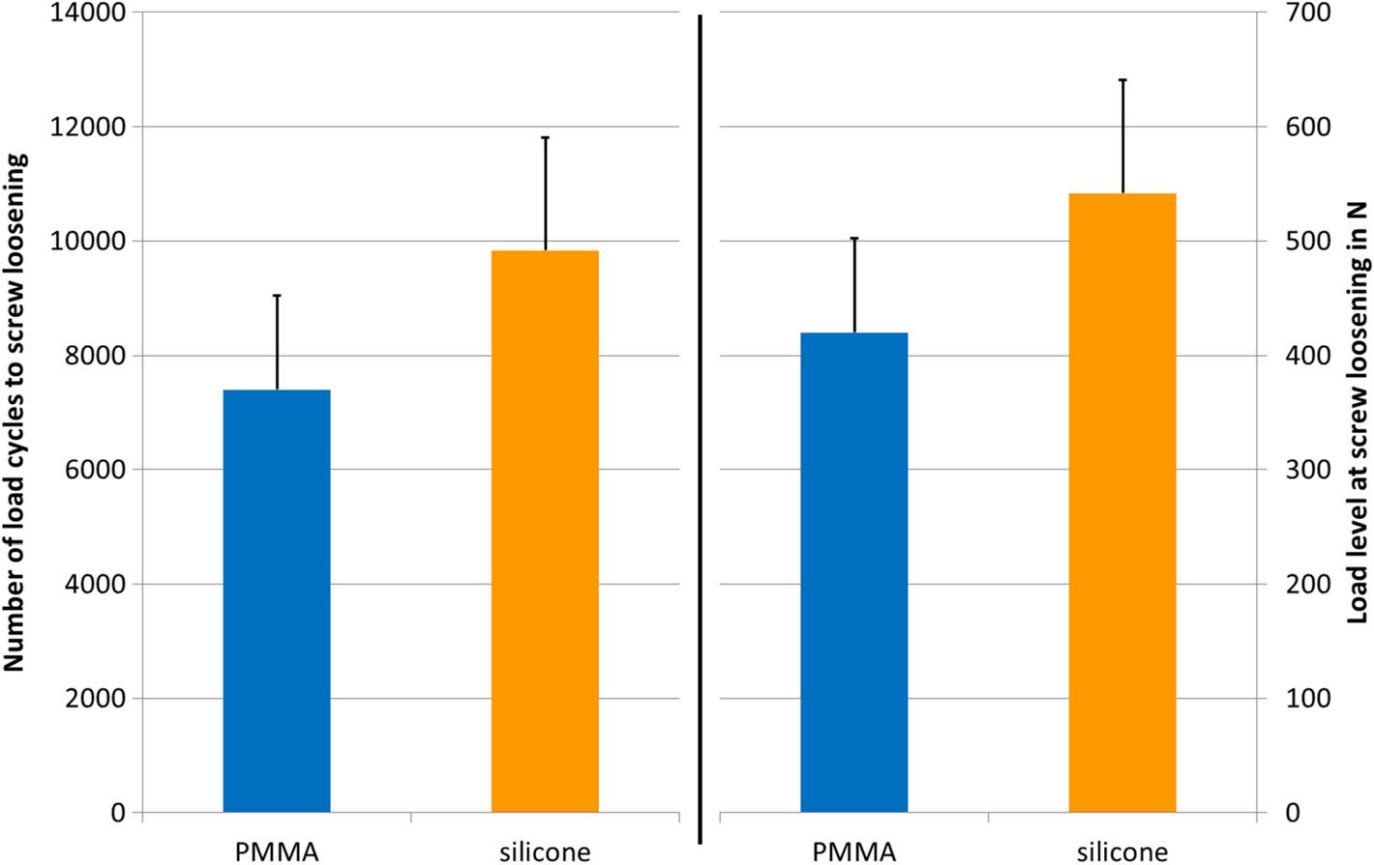
Number of load cycles until screw loosening and corresponding load level. The graph shows the mean and standard deviation

The mean number of load cycles until loosening was 7401 ± 1645 for the in situ PMMA-augmented screws and 9824 ± 1982 for the balloon cavity silicone-augmented screws (*P* = 0.012). With the stepwise increasing load magnitude, these cycle numbers correspond to a mean load level of 420 ± 82 N for in situ PMMA-augmented screws and 542 ± 99 N for balloon cavity silicone-augmented screws (Fig. 3).

In seven out of nine vertebrae, the screws augmented with silicone outperformed the screws augmented with PMMA in terms of load cycles and load magnitude until loosening.

### Pullout test

**Fig. 4:**
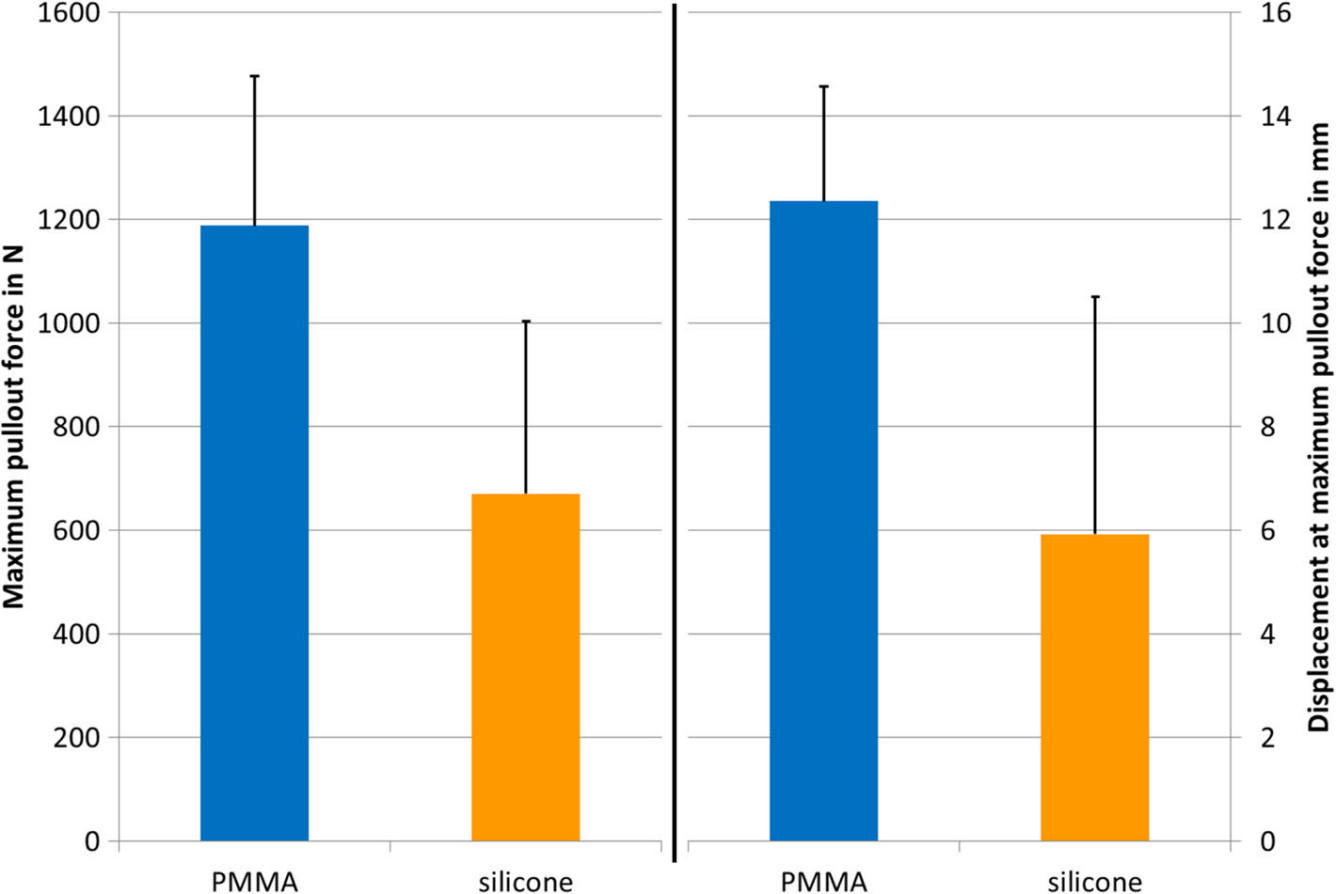
Maximum pullout force and displacement at maximum pullout force (pullout testing was performed after cyclic loading testing). The graph shows the mean and standard deviation

The mean maximum pullout force after loosening was 1189 ± 288 N for in situ PMMA-augmented screws and 671 ± 332 N for balloon cavity silicone-augmented screws (*P* = 0.003) (Fig. 4). The mean displacement at which maximal pullout force was recorded was 12.3 ± 2.2 mm for PMMA-augmented screws and 5.9 ± 4.6 mm for silicone-augmented screws (*P* = 0.002).

For balloon cavity silicone-augmented screws, the main failure mode (seven of nine) during the pullout test was axial pullout of the screw with the silicone cloud still bonded to the trabecular structure while only two specimens failed by pedicle fracture. For in situ PMMA-augmented screws, the main failure mode (eight of nine) during pullout testing was pedicle fracture with the PMMA cement cloud still attached to the screw while only one specimen failed by axial screw pullout.

## Discussion

Several biomechanical studies have shown that, compared to standard non-augmented pedicle screws, augmentation of the screw with PMMA cement can significantly improve screw anchorage in patients with reduced bone quality, independent of the augmentation technique [5, 35, 36]. Therefore, the hypothesis of the present study was that the novel silicone augmentation with prior balloon cavity creation will be non-inferior to in situ augmentation using PMMA cement, which is currently regarded as the gold standard for the improvement of pedicle screw anchorage in several countries [37–39]. Under physiological cyclic loading in cranio-caudal direction, the novel silicone augmentation sustained a significantly higher number of load cycles and load magnitude until loosening than standard PMMA augmentation. In the pullout test after the cyclic loading, the predominant failure mode of in situ PMMA-augmented screws was a fracture of the pedicle with the cement still attached to the screw. In contrast to this, balloon cavity silicone-augmented screws predominantly failed by the pullout of the screw through the pedicle with the augmentation material still attached to the trabecular bone.

The test setup and loading protocol used in the present study consisted of a cyclic cranio-caudal loading with a superimposed bending moment and thus resembles the physiological loading of pedicle screws reported in “in vivo” measurements of patients with an instrumented internal fixator [40–42] much more closely than a pullout test. The cyclic load protocol with a stepwise increasing load magnitude was designed to investigate implant anchorage in reduced bone quality and enables the investigation of implant anchorage for a wide stiffness range in reduced bone quality under cyclic loading [35, 43, 44].

For “in vivo” measurements with an instrumented internal fixator, Rohlmann et al. showed that pedicle screws are mainly loaded axially in cranio-caudal direction and can be superimposed with a small bending moment. In daily activities of the patients, the peak axial forces were reported to range from 100 to 250 N [41, 42]. Using a setup and load protocol similar to that of the present study, Bostelmann et al. reported pedicle screw loosening loads for three different PMMA augmentation techniques ranging from 415 to 453 N, while the non-augmented control only reached 239 N [35]. Hence, they noted that in patients with reduced bone quality, pedicle screws might be loaded during daily activity beyond their loosening threshold. However, with screw augmentation, the load-bearing capacity of pedicle screws can be increased well beyond the load magnitudes occurring during daily activities. The findings of the present study for the in situ PMMA augmentation (420 N) are comparable to the loosening load magnitude reported by Bostelmann et al. for different PMMA augmentation techniques [35], while the balloon cavity silicone augmentation even outperformed the in situ PMMA augmentation.

The post-cycling pullout force of silicone-augmented screws was lower than that of PMMA-augmented screws but was still of higher magnitude than the values reported by Liu et al. for non-augmented and non-cycled screws in osteoporotic (528 N) and severely osteoporotic (358 N) vertebrae [45]. The post-cycling pullout force of PMMA augmented, however, was comparable to the ones reported by Liu et al. for PMMA augmentation (2 ml) without prior cyclic loading [45]. This indicates that the high pullout forces after PMMA augmentation can be attributed to the extensive structural damage caused by pulling the screw with the PMMA still attached through the pedicle, no matter whether the screw is loose or not.

A limitation of this study is that two different augmentation techniques and two different augmentation materials were used. Therefore, it is not possible to decisively attribute the better performance of the balloon cavity silicone augmentation to the material or the technique. Most likely, it is a combination of both. Another limitation is that the study was conducted on cadaver specimens and therefore cannot take account of any biological factors such as bone remodeling and osteointegration, as well as PMMA aging and volumetric shrinkage.

In a comparative study on PMMA screw augmentation techniques, Becker et al. reported no difference in screw anchorage between the augmentation techniques, vertebroplasty, kyphoplasty, and in situ augmentation technique [5]. The present experiment compared two different strategies to enhance pedicle screw anchorage. These two strategies encompassed two different augmentation materials and two different augmentation techniques. Both materials were applied with the technique that best highlights its mechanical properties. The stiffness of PMMA is higher than that of the bone, and during in situ augmentation, the material interdigitates with the trabecular structure and thereby reinforces and interlocks with the trabecular structure [46]. In contrast to that, the stiffness of the self-curing elastomer (VK100) was engineered to resemble the bulk stiffness of trabecular bone and not the stiffness of a single trabecula. Therefore, a cavity was created and filled with silicone in order to benefit from its material properties.

## Conclusion

Pedicle screw augmentation with balloon cavity creation and self-curing elastomeric silicone represents a valuable alternative to PMMA augmentation and resulted in superior pedicle screw anchorage under cyclic cranio-caudal loading. Pullout forces after cyclic loading were higher for in situ PMMA-augmented screws and showed a different failure mode. Using an alternative silicone-based augmentation material, however, might also necessitate a modification of the conventional in situ augmentation technique.
